# A science-based product regulation: the time has come to reduce toxic emissions to reduce harm

**DOI:** 10.3389/fpubh.2025.1687986

**Published:** 2025-10-13

**Authors:** Michael Kunze, José Roberto Santin, Ursula Kunze

**Affiliations:** ^1^Center of Public Health, Department of Social Medicine, Medical University of Vienna, Vienna, Austria; ^2^Post-graduation Program in Pharmaceutical Sciences, Universidade do Vale do Itajaí (UNIVALI), Itajaí, SC, Brazil

**Keywords:** tobacco, tobacco product, toxicity, regulation, FCTC

## Abstract

Tobacco control has focused on reducing use, with little emphasis on regulating product toxicity. Articles 9 and 10 of the World Health Organization's Framework Convention on Tobacco Control (FCTC) offer a mechanism to reduce harm by limiting toxic emissions, but implementation has stalled. A science-based regulatory framework is needed to set emission thresholds for toxicants. Inspiration can be found from other regulated sectors, and the initial focus should be on nine priority toxicants strongly linked to tobacco-related disease. An adaptive, evidence-based approach can complement existing strategies and accelerate harm reduction for more than 1 billion people who still smoke. The 11th FCTC Conference of the Parties in 2025 presents an opportunity to revisit the development of a toxicity reduction strategy.

## 1 Introduction

Tobacco use, primarily in the form of smoked tobacco, remains one of the leading causes of preventable death worldwide, responsible for over 7 million deaths annually according to the WHO, and it generates enormous costs for health systems. The global fight against tobacco-related harm has long relied on measures such as tax-driven price increases, advertising restrictions, health warnings, and smoke-free policies that aim to reduce consumption by minimizing initiation of use and encouraging cessation (1).

Tobacco product regulation, specifically regulation of product emissions to reduce the risk of disease by reducing exposure to toxicants, has been largely overlooked, seemingly too complex to deal with and with little public health upside. Most nicotine use has traditionally occurred through smoking (i.e., inhaling smoke from burning tobacco), a means of consumption where achieving meaningful and broad toxicant reductions is virtually impossible. Hence, reducing tobacco-related harm could only be achieved by decreasing prevalence of the primary form of tobacco and nicotine use, which is smoking. Despite controversies around novel and emerging tobacco and nicotine products, their emergence provides an opportunity to reduce tobacco-related harm not only for those who stop smoking but also for those who are unable or unwilling to abstain from nicotine.

As articulated in the WHO's Tobacco Product Regulation: Basic Handbook (2), the key factors determining the harm caused by tobacco products are their attractiveness, addictiveness, and toxicity. While the appeal and dependence potential of a product impact how many people use a given tobacco product, it is the toxicity—the harmful chemicals released during product use—that directly impacts the health outcomes resulting from the use of such a product:

“If products are made less appealing and more difficult to use, fewer people will begin or continue using tobacco products. If tobacco products are made less addictive […], the amount and frequency of use can be expected to decrease. If overall exposure to tobacco product toxicants is reliably lowered, population harm may be reduced even if large numbers continue to use these products.”

For example, a less toxic product that is used by many people can result in lower levels of morbidity and mortality than a far more toxic product that is used by fewer people (3, 4). In the case of cigarettes, we are faced with a deadly combination of high levels of use and high levels of toxicity.

More than 20 years ago, Kozlowski et al. (5) articulated the need to consider this risk/use equilibrium as follows:

“To the extent use rises faster than risk is decreased, public health will be increasingly disadvantaged. To the extent risk is decreased faster than use rises, public health will be advantaged… For example, if 100 individuals used a product with full danger (for example, killing 100% of users), 10 times that number (1,000 individuals) would need to use a product that had 90% decreased danger, to achieve an equal health problem (100 dead in each instance) … If danger is 0.1%, use would have to increase by 1,000 times to produce a problem of the same magnitude as the full risk product.”

## 2 Policy options

In the field of tobacco policy, the WHO's Framework Convention on Tobacco Control (FCTC), adopted in 2003 and in force since 2005, provides a comprehensive framework with the aim of reducing tobacco-related death and disease by promoting evidence-based tobacco control measures (6).

Articles 9 and 10 of the FCTC regulate content, emissions, and tobacco product disclosures, thereby providing a tool through which harm from tobacco use can be reduced. As the partial guidelines for the implementation of Articles 9 and 10 state, “tobacco product regulation has the potential to contribute to reducing tobacco-attributable disease and premature death by reducing the attractiveness of tobacco products, reducing their addictiveness (or dependence liability) or reducing their overall toxicity” (7). However, the partial guidelines have not provided any guidance with respect to the regulation of harmful constituents and emissions even though this was identified by countries as a priority as far back as the first meeting of the FCTC Conference of the Parties (CoP) in 2006 (8). While national regulatory authorities have the autonomy to implement measures to regulate toxicant emissions, the Parties to the FCTC have struggled to fulfill their obligation to “disclose information about the toxic constituents and emissions of tobacco products to the public in a meaningful way” without this guidance (7). While advances were made in identifying priority toxicants and in validating relevant testing methods, work has focused almost exclusively on attractiveness and addictiveness reduction, and progress on a fundamental toxicity reduction strategy has stalled. The WHO has yet to deliver on a decision made during the 5th CoP meeting requesting a comprehensive report on measures likely to reduce toxicity of tobacco products (9).

Indeed, the proceedings of the Working Group on Articles 9 and 10 were suspended during the 8th meeting of the CoP in 2018 (10). While an expert group was formed instead, it also failed to provide any guidance with respect to reducing harmful constituents and emissions. The matter is scheduled for deliberation at the 11th meeting of the FCTC CoP in November 2025 (11). This represents a unique opportunity to resume this important work, especially in the context of novel and emerging tobacco and nicotine products, provided that any future work is led by the Parties through CoP mechanisms to ensure transparency, legitimacy, and alignment with the Convention's governance structure.

The lack of progress reflects both technical and governance challenges. The Working Group on Articles 9 and 10 was suspended at CoP8 due to disagreements among Parties and the absence of a clear Party-agreed workplan. Even the U.S. FDA, an agency with broad authority and expertise to implement product standards, has not successfully implemented a single toxicant-based standard to date, illustrating the difficulty of translating regulatory authority into action. The EU Tobacco Products Directive requires ingredient and emission reporting, but its scope is limited and has not yielded binding toxicant thresholds, apart from tar, nicotine and carbon monoxide ceilings for cigarettes (12). These experiences highlight gaps but also opportunities for renewed action.

The Parties should consider developing an effective and evidence-based framework analogous to the regulatory practices observed in other sectors. In the chemicals industry, the European Union's REACH regulation sets out substance-specific thresholds based on comprehensive toxicological data (13). Similarly, the European vehicle emission standards impose progressively stricter limits on pollutants based on the latest scientific evidence and technological feasibility (14). In the food sector, additives are regulated based on safety assessments and maximum permissible levels, with periodic reviews that ensure the regulations remain aligned with current scientific knowledge (15).

## 3 Policy implications

The policy implications of adopting such an approach in the tobacco sector are profound. An emissions-based classification system would not only allow regulators to establish and regulate tobacco products based on science-based performance benchmarks, but it could also promote greater understanding of the risk profile of different tobacco and nicotine products with the potential benefit of discouraging the use of the most harmful products. For example, with clear quantitative performance standards in place, product labeling could reflect the level of harm, as is the case today in many countries with respect to energy labels (ranging from household appliances to vehicles to houses).

It is imperative that such a framework is built upon rigorous toxicity and emission studies. The technical foundation for such an approach involves several key elements, including agreement on the list of toxicants that should be measured with due consideration to the way these products are used (i.e., inhaled vs. oral route of delivery), development of standardized measurement methods, and finally setting actual thresholds.

The WHO is well-equipped to advance work in this area and to support countries in its implementation—both through its global network of laboratories specializing in testing tobacco products (TobLabNet), and its TobReg advisory group of international experts in product regulation. However, the policy direction must remain with the Parties to safeguard representativity and avoid unilateral expansion of scope.

Initially, the nine priority toxicants (acetaldehyde, acrolein, formaldehyde, benzene, 1,3-butadiene, carbon monoxide, benzo[a]pyrene, NNK, NNN) most directly associated with carcinogenicity, cardiovascular and pulmonary toxicity could be selected to establish a benchmark that, if met, would serve as a tool to distinguish the most harmful tobacco products from those that are likely to pose lower health risks. These are the same nine toxicants recommended to be reduced in cigarettes almost 20 years ago by TobReg (16).

While the nine priority toxicants remain central for combustible cigarettes, non-combustible products such as heated tobacco, e-cigarettes, and oral nicotine pouches require adapted toxicant lists reflecting their distinct toxicant profile and exposure pathways. For heated tobacco, the nine priority toxicants would initially be sufficient to distinguish the most harmful combusted tobacco products from lower-risk non-combusted products. Standardized testing methods already exist through ISO, CORESTA, and WHO TobLabNet for some constituents, but infrastructure investment in accredited laboratories will be essential. A classification system could be operationalized through a labeling approach analogous to energy efficiency ratings, enabling consumers and regulators to differentiate between higher- and lower-emission products.

It is important to recognize that any future work by the FCTC in this area must be Party-led under the governance of the CoP, with WHO and TobReg providing critical technical input. Embedding clear governance structures will help prevent the stalemates that have previously stalled progress.

It is critical that this framework is not static; as product designs evolve, testing methods must be periodically reviewed and updated to reflect the latest scientific knowledge. This adaptive approach is already employed in other sectors, such as vehicle emissions, where standards are regularly revised. As new products and scientific data emerge, regulatory thresholds can be updated, creating a dynamic system that evolves in tandem with technological advancements and improved understanding of product toxicity. This continuous feedback loop would further promote public health improvements. The potential for public health improvements of such a framework could be even greater than those achieved through the displacement of smoked tobacco products with a low-toxicant smokeless tobacco product called snus in Sweden (17).

## 4 Conclusion

In conclusion, as the tobacco and nicotine market continues to evolve, the need for a science-based regulatory framework that reflects this evolution has never been greater. Product regulation has been an underutilized tool that has great potential to reduce morbidity and mortality caused by tobacco use. An approach based on robust toxicity and emission assessment, with evolving quantitative performance standards, can create a dynamic, adaptive system that protects public health.

Today, such an approach is a public health necessity: the world is not only on track to miss the 2025 global target of a 30% reduction in tobacco use from the 2010 baseline (18), but it is also on track to see more than 1.2 billion people still smoking in 2050 (19).

The existing approach that has only focused on reducing tobacco use has largely been unable to reduce prevalence at a pace that would offset population growth (20). By committing to an evidence-driven regulatory strategy focused on the broader goal of reducing health and societal harm arising from nicotine use through regulation of harmful emissions, the global tobacco control community can ensure that future policies are effective and adaptable to emerging challenges, safeguarding public health for generations to come. The 11th Conference of the Parties, marking the 20th anniversary of the entry into force of the FCTC, could serve as an important milestone for putting in place the foundations for this approach.
